# Synergistic Protective Effect of Curcumin and Resveratrol against Oxidative Stress in Endothelial EAhy926 Cells

**DOI:** 10.1155/2021/2661025

**Published:** 2021-09-03

**Authors:** Xian Zhou, Sualiha Afzal, Yan-Fang Zheng, Gerald Münch, Chun Guang Li

**Affiliations:** ^1^NICM Health Research Institute, Western Sydney University, Locked Bag 1757, Penrith, NSW, Australia; ^2^School of Medicine, Western Sydney University, Campbelltown, NSW 2560, Australia; ^3^College of Pharmacy, Fujian Key Laboratory of Chinese Materia Medica, Fujian University of Traditional Chinese Medicine, Fuzhou 350122, China

## Abstract

Curcumin (C) and resveratrol (R) are two well-known nutraceuticals with strong antioxidant activity that can protect cells from oxidative stress. This study aims to investigate the synergy of CR combinations in protecting human endothelial EAhy926 cells against H_2_O_2_-induced oxidative stress and its related mechanisms. C and R as individual compounds as well as CR combinations at different ratios were screened for their protective effects against H_2_O_2_ (2.5 mM) induced cell death assessed by cell viability assays. The synergistic interaction was analysed using the combination index model. The effects of optimal CR combinations on caspase-3 activity, ROS level, SOD activity, NAD cellular production, expression of Nrf2 and HO-1, and Nrf2 translocation were determined. CR combinations produced a synergistic protection against that of H_2_O_2_-induced changes in cell viability, caspase-3 activity, and ROS production. The strongest effect was observed for CR with the ratio of 8 : 2. Further experiments showed that CR 8 : 2 exhibited significantly greater effects in increasing Nrf2 translocation and expressions of Nrf2 and HO-1 proteins, as well as SOD activity and total cellular NAD production, than that of C or R alone. The findings demonstrate that combination of C and R produced a strong synergy in activity against H_2_O_2_-induced oxidative stress in EAhy926 cells. The mechanism of this synergy involves the activation of Nrf2-HO-1 signaling pathway and promotion of antioxidant enzymes. Further studies on CR synergy may help develop a new combination therapy for endothelial dysfunction and other conditions related to oxidative stress.

## 1. Introduction

Cardiovascular diseases (CVD) are the leading cause of death worldwide and the incidence remains increasing lately in developing countries [1]. Oxidative stress is the major and independent risk factor of CVD. It can lead to endothelial dysfunction, which triggers the depletion of endothelial nitric oxide synthase (eNOS) and vascular constriction leading to the development of atherosclerosis. Oxidative stress, manifested as an overproduction of reactive oxygen species (ROS), plays a central role in mediating various signaling pathways contributing to vascular inflammation from the early stage of the accumulation of fatty streaks to ultimate plaque rupture [2]. In addition, other traditional risk factors of CVD such as smoke, aging, and arterial hypertension also contribute to the oxidative damage to cellular macromolecules such as DNA, lipids, and proteins and endothelial dysfunction [3]. Thus, oxidative stress remains an important therapeutic target for cardiovascular diseases, particularly in the early stage of atherosclerosis. Nutraceuticals that are rich in antioxidant components have been shown to be beneficial to lower the burden of oxidative stress in CVD [4].

Curcumin (C) and resveratrol (R) are very popular nutraceuticals that have been demonstrated to have strong antioxidant properties. Curcumin is the key bioactive compound from turmeric (*Curcuma longa* L.), which has been widely used for centuries as a traditional and indigenous medicine for inflammatory-related conditions [5]. Curcumin has been shown as a potent scavenger of a variety of free radicals including ROS, hydroxyl radicals, and nitrogen dioxide radicals [6]. Many studies have investigated its potential therapeutic action in renal protection [7], myocardial ischemic damage [8], and ethanol-induced liver injury [9] where the oxidative stress is the main pathological component [10]. Resveratrol is a phenolic micronutrient compound found in grapes, peanuts, and red wine. Modern scientific studies have linked resveratrol to age-related diseases and disability through its action in the induction of sirtuin 1 and DNA repair [11]. In addition, both compounds have been shown to regulate the antioxidant response through the induction via nuclear factor erythroid factor 2-related factor 2 (Nrf2), a critical transcription factor that regulates the expression of over 1000 genes in the cell under normal and (oxidative) stressed conditions [12].

It has been recognized that combination drug therapy using two or more components in a fixed-dose ratio may render synergistically enhanced efficacy and/or reduced toxicity. It has been gradually adopted in drug development to overcome the obstacles of those pharmaceutical agents with limited efficacy or a narrow therapeutic window. C and R have been found to act on multiple key regulators related to antioxidant in the cells and can potentially produce a synergistic effect. There is evidence showing a synergistic interaction of C and R in anticancer properties [13–23]. Several studies have also investigated the combined antioxidant activities of C and R. Zaky et al. showed that combined C and R (both in 200 mg/kg) exhibited higher activity in attenuating aluminium-induced neuroinflammation in rats compared to monotherapy, which was partly attributed to the enhanced antioxidant activity [reduced total lipid peroxidation, restored glutathione (GSH), glutathione S-transferase and superoxide dismutase determination (SOD)] [24]. AlBasher et al. investigated the combined activity of C (200 mg/kg) and R (10 mg/kg) in fipronil-triggered oxidative damage in male albino rats and found that the combination synergistically elevated tissue oxidative injury and restored the antioxidant enzymes including GSH and SOD [25]. Similar effects were also seen for C (50 mg/kg.b.w) and R (25 mg/kg.b.w) in obese diabetic rats fed with high fat diet which was related to their synergistic antioxidant activity [26].

Previous studies indicated a plausible interaction of C and R in antioxidant activity, although only specific compositions were tested, and the relevant mechanisms involved remain unclear. This study aims to investigate the interaction of C and R in protecting endothelial cells against oxidative stress and relevant mechanisms on modulating key oxidative stress-related cellular regulators and antioxidant enzymes. The outcome helps to understand the synergy between these two molecules and enhance the application of these natural compounds as nutraceuticals in the prevention and treatment of CVD.

## 2. Materials and Methods

### 2.1. Cell Culture and Drug Treatments

Human cardiovascular endothelial cell line (EAhy926) was purchased from ATCC, USA (ATCC®CRL-2922™). It was cultured in DMEM/Ham's F12 (Lonza, Australia) and supplemented with 10% fetal bovine serum (FBS) and 100 U/mL of penicillin-streptomycin (Gibco BRL, Australia). The stable human mammary MCF7-derived reporter cell line (transfected with Nrf2) (AREc32) was obtained from Professor Gerald Münch in School of Medicine, Western Sydney University. It was cultured in DMEM (Lonza, Australia) supplemented with 10% FBS and 100 U/mL of penicillin-streptomycin (Gibco BRL, Australia). Both cell lines were grown in a 5% CO_2_-humidified incubator at 37°C.

The reference compounds of C and R were purchased from Chengdu Biopurify (China) and Sigma-Aldrich (Australia), respectively. They were both dissolved in dimethyl sulfoxide (DMSO, final concentration was 0.1%). The combinations of C-R were prepared by mixing the same concentrations of C and R (both at 50 mM) in DMSO in different ratios by volume (1 : 9, 2 : 8, 3 : 7, 4 : 6, 5 : 5, 6 : 4, 7 : 3, 8 : 2, 9 : 1, *v*/*v*). Then, they were diluted with media and subjected to the cells with serial dilutions.

### 2.2. Cell Viability Assessment by Alamar Blue and MTT Assay

To investigate the protective effects of C, R, and CR on endothelial cell toxicity caused by hydrogen peroxide (H_2_O_2_), we followed the methods of Zhou et al. [27]. EAhy926 cells (1 × 10^6^ cells/mL) were incubated with increasing concentrations (1.65–50 *μ*M) of C, R, or CR for 1 h before the stimulation of H_2_O_2_ (Sigma-Aldrich, Australia) at 2.5 mM to induce the cell death. After 12 h, the cell supernatant was replaced with Alamar Blue (10 *μ*g/mL) or MTT (0.15 mg/mL) in PBS for 2 h. The fluorescent absorbance of Alamar Blue was measured at 540 nm excitation and 590 nm emission using a microplate reader (BMG LABTECH FLUOstar OPTIMA, Mount Eliza, Victoria, Australia). The supernatant of the cells incubated with MTT was discarded and replaced with DMSO. The absorbance was then measured at 540 nm using a microplate reader (BMG LABTECH FLUOstar OPTIMA, Mount Eliza, Victoria, Australia).

### 2.3. Caspase-3 Activity

The cell apoptotic level of EAhy926 cells with or without treatments was measured by the fold change of caspase-3 cellular protein level followed by Zhou et al. [27]. EAhy926 cells were seeded in 96-well flat-bottom cell culture plates and incubated for 24 h until confluency. After the treatment period of 1 h, H_2_O_2_ (2.5 mM) was added to the cells for another 4 h to induce apoptosis. The cells were then centrifuged at 250 ×g for 10 min to remove the cell supernatant and the total protein was collected by incubating the cells with lysis buffer on ice for 10 min and centrifugation at 10,000 ×g for 1 min. The total protein was determined by a Pierce BCA Protein Assay Kit (Thermo Fisher Scientific, Australia) and diluted to 1 mg/mL. The quantitative caspase-3 activity assay was conducted using the caspase-3 colorimetric commercial kit (Abcam, Australia) according to the manufacturer's protocol. The plate was incubated at 37°C for various time points, and the absorbance was read on a microplate reader (BMG LABTECH FLUOstar Optima, Mount Eliza, Victoria, Australia) at a wavelength of 410 nm.

### 2.4. ROS Assay and Confocal Microscopy

The intracellular level of oxidative stress with or without treatments was measured by ROS levels according to the protocol of cellular ROS assay kit cited in [27]. EAhy926 cells (2.5 × 10^5^ cells/mL) were seeded on a black 96-well cell culture plate and allowed to reach confluence overnight. The cells were then washed once with 1X assay buffer (from the kit) and stained with 2′,7′-dichlorofluorescin diacetate (DCFDA) (20 *μ*M) at 100 *μ*L per well for 45 min at 37°C in the dark. The plate's initial absorbance was recorded under a microplate reader as *A*0 (BMG LABTECH FLUOstar Optima, Mount Eliza, Victoria, Australia) with excitation at 455 nm and emission 535 nm in fluorescence mode. After washing with ice-cold PBS, the cells were treated with C, R, or C-R at increasing concentrations (3.13–25 *μ*M). After the incubation for 1 h, the absorbance was measured again under the same setting and recorded as *A*1. The fold change of ROS was calculated as *A*1 normalised to its corresponding *A*0 (*A*1/*A*0). tert-Butyl hydroperoxide (tBHP) was used as a positive control in this assay.

EAhy926 cells were grown in an 8-chamber slide (Thermo Fisher Scientific, Australia) to allow confluency overnight. After washing with ice-cold PBS, and cells in the chamber were stained with DCFDA (20 *μ*M) for 45 min at 37°C in the dark. The staining solution was then replaced with the treatment of C, R, or CR 8 : 2 for another 1 h and subjected to inverted Leica TCS SP5 laser scanning confocal microscope for imaging. The filter was set at 488 for ROS fluorescein.

### 2.5. Nrf2 Translocation by Immunofluorescence Staining and Protein Expression by Luciferase Assay

EAhy926 cells (20,000 cells per chamber) were seeded in an 8-chamber slide (Thermo Fisher Scientific, Australia) overnight and were treated with serially diluted CR 8 : 2 (6.25–25 *μ*M) for 24 h. The cells were then washed with cold PBS and fixed with 4% paraformaldehyde for 15 min, followed by adding Triton X-100 (0.1%) for 15 min. After blocking with 1% BSA for 1 h, the cells were coincubated with rabbit mAb Nrf2 (1 : 50, Cell Signaling Technology, USA) for 1 h and washed with PBS for 3 times and incubated with goat-rabbit IgG conjugated with Alexa Fluor 594 (red) 1 : 1000. After washed for another 3 times, the chamber slide was stained with antifade mounting medium with DAPI (Sigma, Australia) and subjected to immunofluorescent imaging using an inverted Leica TCS SP5 laser scanning confocal microscope.

AREc32 cells were seeded at a density of 1.0 × 10^6^ cells/mL in 96-well plates. The Nrf2 total cellular protein production was detected by luciferase assay with optimization [28]. After 24 hours' incubation, the cells were treated with tert-butyl hydroquinone (tBHQ) (positive control), CR combinations, or medium only (negative control). The cells were incubated with Triton lysis buffer (tris HCl: 1.705%, tris base: 0.508%, 5 M NaCl: 1.5%, 1 M MgCl_2_: 0.3%, Triton X-100 pure liquid: 0.75%) for 20 min at −20°C and mixed with luciferin buffer (D-luciferin 30 mg/mL: 0.525%, DTT 1 M: 3%, coenzyme A 10 mM: 1.5%, ATP 100 mM: 0.45%) for 100 *μ*L per well. The absorbance was measured within 30 minutes at an excitation wavelength of 488 nm and an emission wavelength of 525 nm. The activation of Nrf2 was calculated by fold compared to the negative control (cells with medium only). Cell viability was presented by percentage relative to the negative control (%).

### 2.6. HO-1 Protein Expression

EAhy926 cells were incubated with serum-free DMEM media (blank), C, R, or CR at 50 *μ*g/mL in T75 cell flasks for 24 hours. The cells were then lysed with RIPA lysis buffer (Thermo Fisher Scientific, Australia) and the protein was collected and quantified using BCA quantification kit (Thermo Fisher Scientific, Australia). Equal amounts of proteins were then separated by SDS-PAGE and transferred to a PVDF membrane. The membrane was blocked by 5% milk and then incubated overnight at 4°C with rabbit polyclonal antibodies against heme oxygenase-1 (HO-1, 1 : 1000, Cell Signaling Technology, USA) and beta-actin (1 : 1000, Cell Signaling Technology, USA), followed by anti-rabbit horseradish peroxidase-conjugated secondary antibodies. Then, the membranes were exposed to Pierce ECL Plus western blot substrate (Thermo Fisher Scientific, Australia). The band intensities of the membranes were quantified by ImageJ, and the control for equivalent protein loading was assessed by anti-*β*-actin antibody.

### 2.7. SOD Activity and Total Cellular Nicotinamide-Adenine Dinucleotide (NAD) Production

EAhy926 cells cultured in the T25 flasks were treated with media with vehicle, C, R, or CR for 4 h, and the cells were harvested and lysed by lysis buffer (0.1 M tris HCl, pH 7.4 containing 0.5% Triton X-100, 5 mM *β*-ME, 0.1 mg/mL PMSF). The protein was collected after centrifugation at 14,000 ×g for 5 min at 4°C. The detection of SOD was conducted using the commercial kit from Sigma-Aldrich (Australia) according to the manufacturer's protocol.

EAhy926 cells were seeded with 1 × 10^5^ cells/mL for 120 *μ*L per well and allowed confluency overnight. The cells were then treated with media with vehicle, C, R, or CR for 24 h. The total cellular NAD production was measured using the NAD/NADH cell-based assay kit (Cayman, Australia).

### 2.8. Synergy Determination and Statistical Analysis

CompuSyn (Biosoft, US) was used to analyse the interaction between C and R. The specific measurement for the combination index (CI) value represents the interaction level, where CI < 1, CI = 1, and CI > 1 suggest synergistic, additive, and antagonistic interactions, respectively. The calculation of CI values was based on Chou-Talalay method and determined by CompuSyn [29]. Briefly, the dose-response curves of individual or combined C and R from the cell viability assay or Nrf2 assays were input to the CompuSyn. The program then generated the CI-Fa (fraction affected level) curve and the relevant statistics regarding the synergistic/antagonistic interactions.

All statistics comparisons were performed using GraphPad Version 8 (US). The significance was analysed by one-way ANOVA test. Data were expressed as mean ± SD with at least three individual experiments (*n* ≥ 3). *p* < 0.05 was considered statistically significant.

## 3. Results

### 3.1. Synergistic Effect of CR on Restoring Cell Viability of EAhy926 Cells

**Figure 1:**
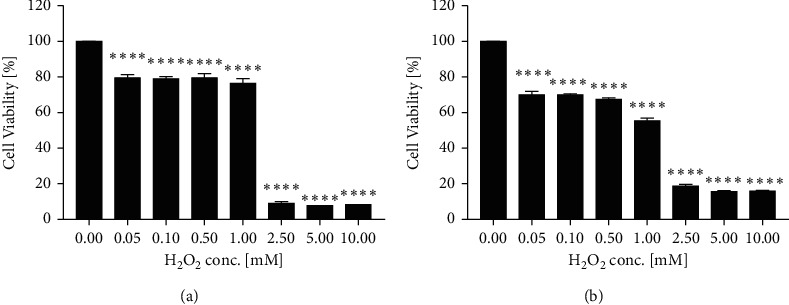
H_2_O_2_ dose-dependently reduced cell viability in EAhy926 cells by Alamar Blue assay (a) and MTT assay (b).^∗∗∗∗^*p* < 0.0001 vs. H_2_O_2_ concentration = 0.00 mM.

**Figure 2:**
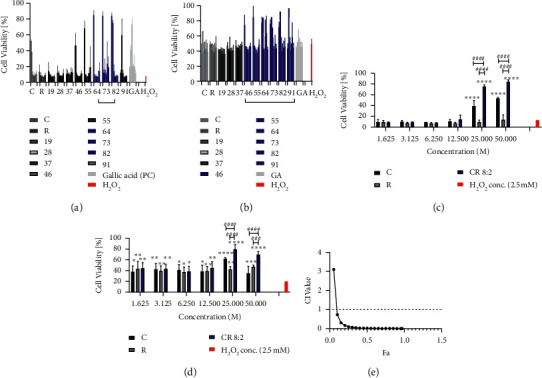
Individual and combined C and R restored H_2_O_2_ impaired cell viability in EAhy926 cells assessed by Alamar Blue and MTT assays (*n* = 3). Pretreatment of C, R, or CR in different ratios (1 : 9, 2 : 8,…, 9 : 1) restored the cell viability of EAhy926 cells against the stimulation of H_2_O_2_ at 2.5 mM assessed by Alamar Blue assay (a). Gallic acid (GA) was used as a positive control in this model. In particular, the effect of CR 8 : 2 in restoring the impaired cell viability was generally greater than that of C or R as assessed by Alamar Blue assay (c). Similar trend was found in the MTT assay that pretreatment of C, R, or CR in different ratios (1 : 9, 2 : 8,…, 9 : 1) restored the cell viability (b), and the effect of CR 8 : 2 in restoring the impaired cell viability was generally greater than that of C or R (d). ^∗∗∗∗^*p* < 0.0001, ^∗∗∗^*p* < 0.001, ^∗∗^*p* < 0.01, ^*∗*^*p* < 0.05 vs. H_2_O_2_ at 1 mM. ^####^*p* < 0.0001, ^###^*p* < 0.001, ^##^*p* < 0.01, ^#^*p* < 0.05 vs. CR 8 : 2 at the same concentration level. The error bars represent the standard deviation of measurements for three samples in three separate sample runs (*n* = 3). Synergistic interaction of CR in restoring cell viability analysed by CI-Fa curve (e).

As shown in Figure 1(a), H_2_O_2_ dose-dependently (0.05–10 mM) reduced the cell viability in EAhy926 cells compared to the untreated cells (cells with media only) with *p* < 0.0001 in Alamar Blue assay. At 2.5 mM, H_2_O_2_ started to reduce the cell viability dramatically to 8.93 ± 1.79%. The positive control, gallic acid (GA), dose-dependently (1.625–50 *μ*M) restored the cell viability impaired by H_2_O_2_ (2.5 mM), with a substantial increase of cell viability to 72.92 ± 8.54% at 12.5 *μ*M. The pretreatment of C significantly restored cell viability at 25 and 50 *μ*M (*p* < 0.0001), whereas R did not show any significant effect. Combinations of CR showed prominent effects in restoring cell viability. As shown in Figure 2(a), high cell viability levels were generally seen in CR combinations with ratios of 6 : 4, 7 : 3, and 8 : 2, which were generally more effective than that of C or R alone. CR combinations with ratios of 4 : 6, 5 : 5, and 9 : 1 also markedly increased cell viability, and the maximum effects were slightly higher than that of C. We have further analysed the effect of CR 8 : 2 combination in comparison to C or R by Alamar Blue assay. As shown in Figure 2(c), CR 8 : 2 significantly restored the cell viability at 25 and 50 *μ*M, and the effects were significantly higher than that of C or R (both *p* < 0.0001).

Similarly, H_2_O_2_ dose-dependently (0.05–10 mM) reduced the cell viability of EAhy926 cells (*p* < 0.0001) in MTT assay, as shown in Figure 1(b). A similar trend of effects for CR combinations was found in the MTT assay (Figure 2(b)). CR combinations with the ratios of 4 : 6, 5 : 5, 6 : 4, 7 : 3, 8 : 2, and 9 : 1 all restored the impaired cell viability, of which combined effects were generally stronger than that of C or R alone. In particular, CR 8 : 2 showed a stronger effect than that of C or R at the concentrations of 25 and 50 *μ*M (*p* < 0.0001, Figure 2(d)). We have applied CI model to determine the synergistic interaction between C and R. CI-Fa curve in Figure 2(e) suggested a strong synergy in most concentration levels (CI < 0.721 when Fa ranged from 0.1 to 1.0). The CI and Fa values of all tested CR combinations in cell viability against H_2_O_2_ in EAhy926 cells assessed by Alamar Blue and MTT assays are shown in Supplementary Material S1.

### 3.2. Enhanced Effect of CR on Inhibiting Caspase-3 Activity

**Figure 3:**
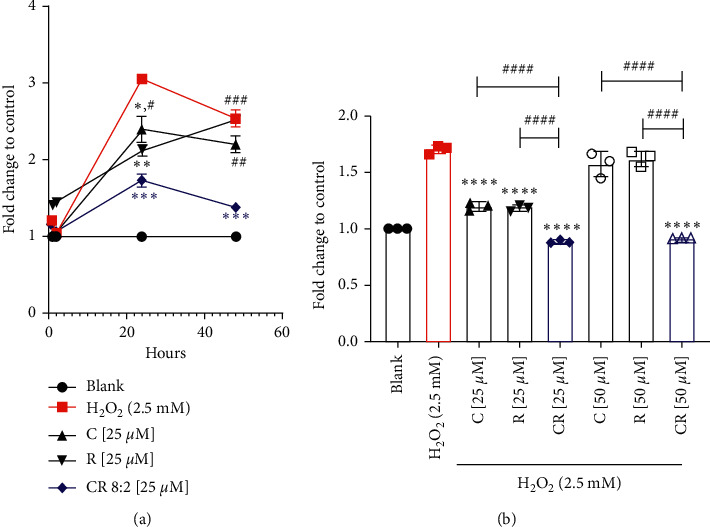
CR exhibited an enhanced effect in inhibiting H_2_O_2_-induced caspase-3 activity in comparison to that of the individual compound. (a) Fold change of caspase-3 activity in different time points. ^###^*p* < 0.001, ^##^*p* < 0.05vs. CR at the same concentration level. ^∗∗∗^*p* < 0.001, ^∗∗^*p* < 0.01, ^*∗*^*p* < 0.05 vs. H_2_O_2_. (b) CR (25 and 50 *μ*M) significantly reduced the fold increase of caspase-3 against H_2_O_2_ in EAhy926 cells (*n* = 3). ^####^*p* < 0.0001 vs. CR at the same concentration level. ^∗∗∗∗^*p* < 0.0001 vs. H_2_O_2_ (2.5 mM). The error bars represent the standard deviation of measurements for three samples collected from three individual experiments in one assay run (*n* = 3).

EAhy926 cells incubated with H_2_O_2_ (2.5 mM) for 24 h caused a significant increase in caspase-3 activity measured at 24 h (*p* < 0.05) compared to the control group, indicating an increased cell apoptotic level. The fold change was decreased slightly at 48 h. As shown in Figure 3(a), C (25 *μ*M) significantly reduced the fold change of caspase-3 at 24 h (*p* < 0.05), suggesting a reduced protein expression at that time. However, the effect was not significant at 48 h. Similarly, R (25 *μ*M) significantly reduced caspase-3 activity at 24 h (*p* < 0.01), but did not cause a significant attenuation at 48 h (*p* < 0.05). Noticeably, significant inhibitory effects were observed with CR 8 : 2 at 25 *μ*M at 24 h (*p* < 0.0001) and 48 h (*p* < 0.001). The fold change of caspase-3 in CR 8 : 2 was significantly lower than that of C or R at both time points, suggesting a stronger effect of CR 8 : 2 in reducing caspase-3 induced by H_2_O_2._ We have specifically analysed the caspase-3 level of C, R, and CR (25 and 50 *μ*M) at 24 h, as shown in Figure 3(b). At 25 *μ*M, C, R, and CR significantly reduced the fold change of caspase-3 (*p* < 0.0001) compared to that of H_2_O_2_ group. However, the effect of CR was stronger than that of C or R (*p* < 0.0001). At 50 *μ*M, only CR was found to significantly inhibit caspase-3, whereas C or R did not show any significant effect. The combined effect remained significantly stronger than that of C or R (*p* < 0.0001).

### 3.3. Synergistic Effect of C-R on Scavenging ROS

**Figure 4:**
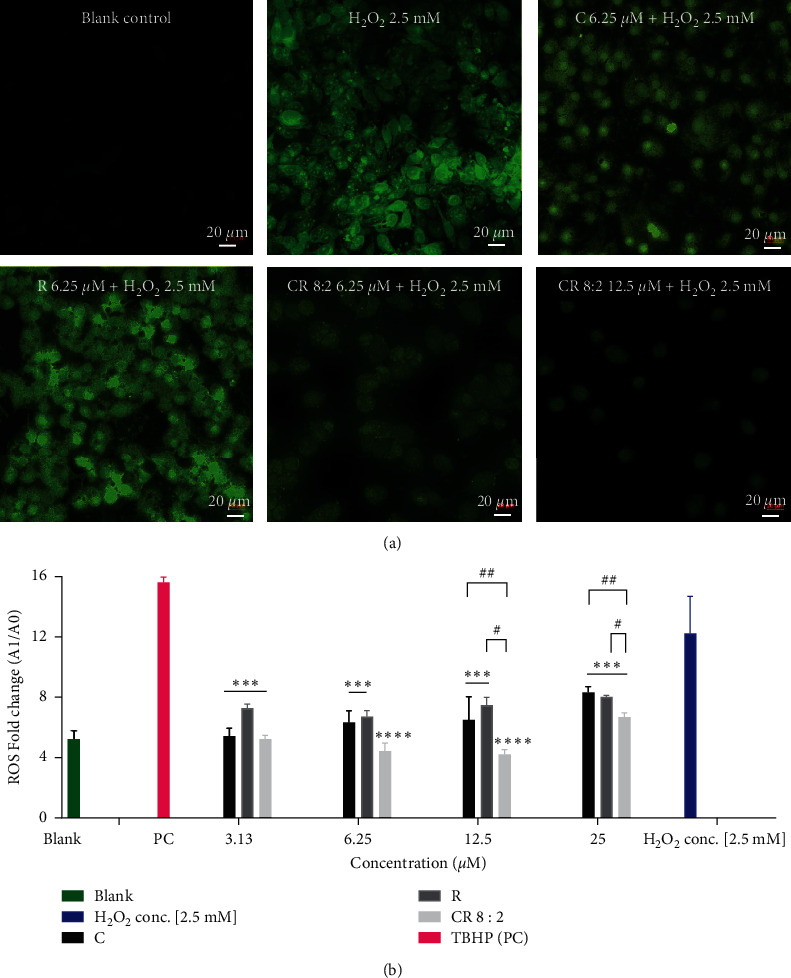
ROS expression in EAhy926 cells pretreated with C, R, or CR 8 : 2 and stimulated with H_2_O_2_ for 4 h. (a) EAhy926 cells were stained with DCFDA (fluorogenic dye) for 1 h and washed and incubated with various treatments for 1 h and stimulated with H_2_O_2_ (2.5 mM) for another 4 h. The cells were subjected to a confocal microscope (20x) for the imaging of ROS detection with the filter set of fluor488 using an inverted Leica TCS SP5 laser scanning confocal microscope. (b) EAhy926 cells were stained with DCFDA (fluorogenic dye) for 1 h and washed and incubated with various treatments for 1 h and stimulated with H_2_O_2_ (2.5 mM) for another 4 h. The ROS amount was quantified by reading the absorbance on a fluorescence plate reader at *Ex*/*Em*  = 485/535 nm. ^∗∗∗∗^*p* < 0.0001, ^∗∗∗^*p* < 0.001 vs. H_2_O_2_. ^#^*p* < 0.05, ^#^*p* < 0.01 vs. C or R at the same concentration level. TBPH was used as PC (positive control) in this assay. The error bars represent the standard deviation of measurements for three samples in three assay runs (*n* = 3).

Figure 4(a) shows that EAhy926 cells stimulated with H_2_O_2_ (2.5 mM) expressed a strong signal of ROS (green fluorescent) compared to that of untreated cells (blank control). Pretreatments of C, R, and CR 8 : 2 (6.25 *μ*M) significantly inhibited ROS expression. In particular, the reduction of ROS in CR 8 : 2 was greater than that of C or R at the same concentration level (6.25 *μ*M). The ROS level was further reduced by CR 8 : 2 at 12.5 *μ*M. Our quantitative analysis of ROS by microplate assay suggested that the fold increase of ROS stimulated by H_2_O_2_ (2.5 mM) for 4 h was 11.78 ± 2.58 compared to the blank control which was 5.22 ± 0.58 (Figure 4(b)). TBHP was used as a positive control in this assay, which boosted the ROS level to 15.62 ± 0.35. Treatment of C, R, and CR all significantly reduced the ROS fold change against H_2_O_2_ (2.5 mM) at 3.13–25 *μ*M (*p* values < 0.001). Furthermore, CR 8 : 2 showed stronger inhibitions of ROS at 12.5 and 25 *μ*M (*p* < 0.0001), in which the reductions were both significantly greater than that of C or R alone at the same concentration level (*p* < 0.05).

### 3.4. Associated Mechanistic Pathways Related to the Synergistic Effect of CR on Oxidative Stress

#### 3.4.1. Activation of Nrf2

**Figure 5:**
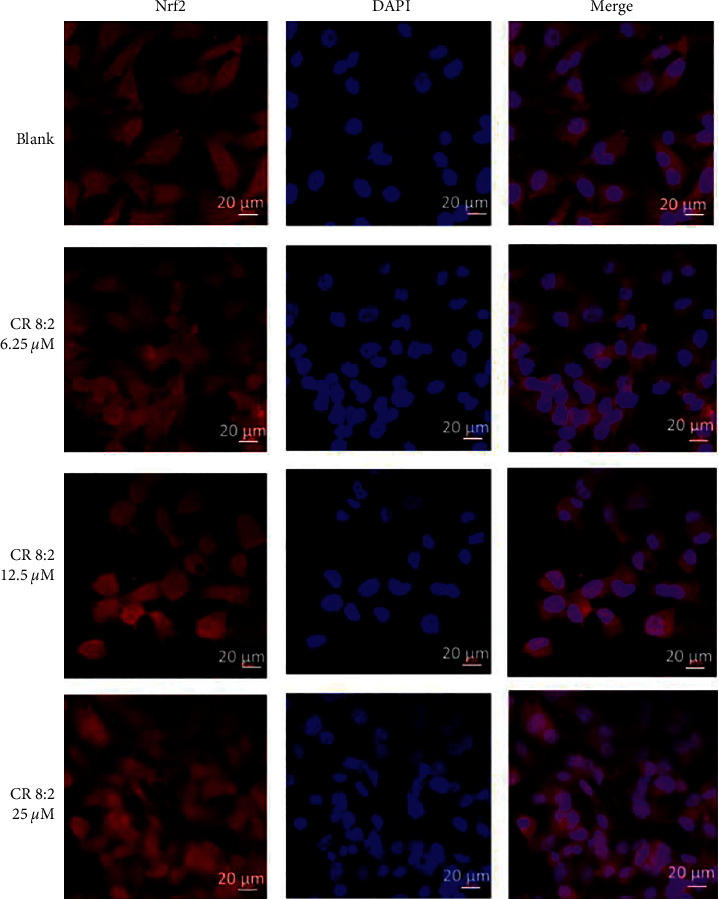
C, R, and CR 8 : 2 induced Nrf2 translocation in EAhy926 cells (*n* = 3). Immunofluorescent analysis was performed with an inverted Leica TCS SP5 laser scanning confocal microscope. The red and blue fluorescent indicates the localization of Nrf2 and nucleus (DAPI), respectively. The scale bars represent 20 *μ*m.

**Figure 6:**
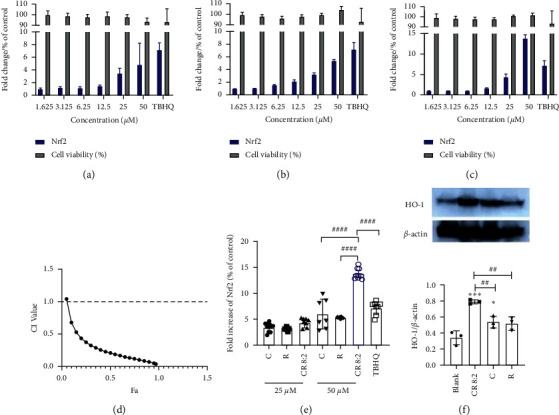
CR 8 : 2 synergistically increased Nrf2 and enhanced the upregulation of HO-1 protein expression (*n* = 3). Nrf2 protein activation of C (a), R (b), and CR 8 : 2 (c) compared to the negative control (untreated cells) measured by luciferase assay with AREc32 cell line. (d) The CI-Fa curve of CR 8 : 2 upregulating Nrf2 as analysed by CompuSyn software. (e) The comparison of Nrf2 fold increase in C, R, and CR 8 : 2 at 25 and 50 *μ*M. (f) Representative western blot images of HO-1 protein and the quantitative expressions following the coincubation of C, R, and C-R (25 *μ*M) in EAhy926 cells measured by western blot analysis. ^∗∗^*p* < 0.01 vs. blank; ^#^*p* < 0.05 vs. C or R at the same concentration level. The error bars represent the standard deviation of measurements for at least three samples in three separate assay runs (*n* = 3).

We investigated if the protective effect of CR combinations on endothelial cells was associated with the activation of the Nrf2 pathway. As shown in Figure 5, Nrf2 was mainly located in the cytoplasm without any stimulation. The coincubation of CR 8 : 2 (6.25–25 *μ*M) dose-dependently increased the nuclear levels of Nrf2 as observed by the overlapped red and blue fluorescent staining, suggesting that CR 8 : 2 induced the Nrf2 translocation. Followed by this observation, we then conducted the Nrf2 luciferase assay to quantify the Nrf2 protein expression by C, R, and CR 8 : 2. As shown in Figures 6(a) and 6(b), C and R dose-dependently increased the fold change of Nrf2 compared to that of the negative control (untreated cells), and the maximum fold changes observed at 50 *μ*M C or R were 5.96 ± 2.87 and 5.29 ± 0.17, respectively. The maximum increase of Nrf2 expression by C or R was not as high as that of the positive control, TBHQ, which increased Nrf2 expression by 7.07 ± 1.27 fold at 12.5 *μ*M. On the other hand, CR 8 : 2 (1.625–50 *μ*M) increased Nrf2 expression in a dose-dependent manner, as shown in Figure 6(c). The maximum fold change was seen at 50 *μ*M (13.74 ± 1.00 fold), which was even more prominent than that of TBHQ (7.07 ± 1.27 fold). CI-Fa curve (Figure 6(d)) revealed that there was a strong synergy in upregulating Nrf2 by CR 8 : 2, with CI values lower than 1 when the total dosages were ranged from 9.30–34.98 *μ*M. We also compared the Nrf2 induction by CR 8 : 2 to that of individual C or R at 25 and 50 *μ*M.  Figure 6(e) revealed that the induction of Nrf2 by CR 8 : 2 (50 *μ*M) was significantly stronger than that of C (*p* < 0.0001) or R (*p* < 0.0001) at the same concentration level and TBHQ (12.5 *μ*M) with *p* < 0.0001.

#### 3.4.2. Increased Protein Expression of HO-1

We then analysed the induction of HO-1 protein, the downstream key protein of Nrf2 pathway, by C, R, or CR 8 : 2, as shown in Figure 6(f). Western blot analysis showed that both C and CR 8 : 2 significantly increased the HO-1 protein expression (*p* < 0.05 and *p* < 0.001, respectively). Although R also showed an increasing trend of HO-1 expression compared to that of untreated cells, the increase did not reach a significance level. In particular, the increase of HO-1 expression by CR 8 : 2 was significantly stronger than that of C or R alone (both *p* < 0.01).

### 3.5. Activation of Antioxidant-Related Enzymes

#### 3.5.1. Increased SOD Activity

**Figure 7:**
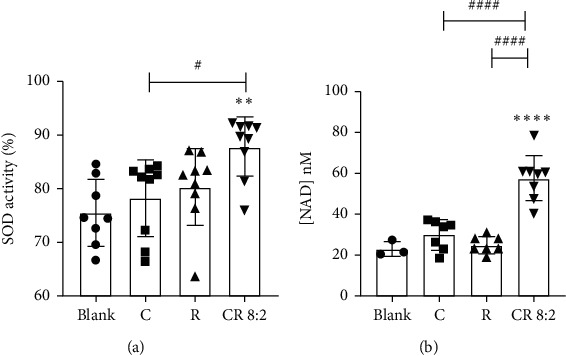
CR 8 : 2 increased cellular SOD (a) and NAD (nM) (b) productions. ^∗∗^*p* < 0.01, ^∗∗∗∗^*p* < 0.0001 vs. blank; ^#^*p* < 0.05, ^####^*p* < 0.0001 vs. C or R at the same concentration level (50 *μ*M). The error bars represent the standard deviation of measurements for over three samples in three separate assay runs (*n* = 3).

As shown in Figure 7(a), C or R alone showed an increasing trend of SOD activity, although it did not reach a statistical significance compared to that of blank (untreated cells). In contrast, CR 8 : 2 significantly increased the SOD activity (87.80 ± 5.53% vs. blank 75.52 ± 6.53%), *p* < 0.01. The increase of SOD activity by CR 8 : 2 was significantly higher than that of C or R (*p* < 0.05).

#### 3.5.2. Total Cellular NAD Production

As shown in Figure 7(b), C or R by itself did not change the NAD production significantly compared to that of the blank control (untreated cells), whereas CR 8 : 2 significantly boosted the NAD production to 57.82 ± 11.23 nM (*p* < 0.0001 vs. blank 21.08 ± 3.63 nM). The increase of NAD by CR 8 : 2 was also significantly higher than that of C or R (both *p* < 0.00011).

**Figure 8:**
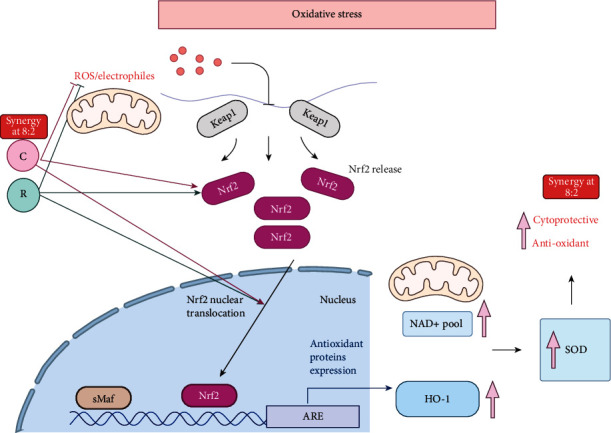
Antioxidant and protective effects of C and R in EAhy926 cells against oxidative stres*s* in vitro. H_2_O_2_ stimulates the ROS which then triggers the Nrf2 release and translocation, which initiates the activation of HO-1, upregulation of NAD^+^ pool, and SOD activity. In EAhy926 cells, C and R both inhibited the ROS expression and increased Nrf2 and HO-1 protein expressions. The combined effect has led to a further promoted inhibition of ROS and increased expression of Nrf2, HO-1, NAD+, and SOD and consequently resulted in a higher survival rate of endothelial cells against the impairment of oxidative stress.

A brief diagram illustrating the synergistic action of CR in protecting endothelial cells against oxidative damage is shown in Figure 8.

## 4. Discussion

Individual treatments of C and R have been extensively studied in many *in vitro* and *in vivo* studies for their antioxidant properties. The present study shows for the first time that combinations of C and R in certain compositions exhibit synergistically enhanced cytoprotective and antioxidant activities in endothelial cells with inhibited ROS levels and activated antioxidant regulators such as Nrf2, HO-1, SOD, and NAD. In particular, the combinational antioxidant effect of CR 8 : 2 was more prominent than that of the single component on multiple key targets related to Nrf2-HO-1 signaling pathway.

Individual C and R have been demonstrated to have a cytoprotective effect against oxidative stress through various mechanisms [30–32]. In line with previous findings [33], our results showed that C exhibited antioxidant activity by scavenging ROS, attenuation of caspase-3 activation, and increased cell survival in H_2_O_2_-treated EAhy926 cells. A study from Guo et al. also showed a similar finding that C significantly reduced apoptosis of EAhy926 cells by reversing the alterations in caspase-3, Bcl-2, and Bax expressions [33]. Such antioxidant and cytoprotective activities of C have been attributed to its unique structure and different functional groups which allow its penetration into the polar medium inside the cells. It can directly scavenge intracellular smaller oxidative molecules such as H_2_O_2_, HO^·^, and ROO^·^ by readily transferring electrons or easily donating H-atom from two phenolic sites [34]. On the other hand, the cardiovascular protective effect of R has been associated with the induction of key endogenous antioxidants including glutathione S-transferase, catalase, and NAD(P)H: quinone oxidoreductase-1 which increase the cellular defence and resistance to oxidative stress [35]. Our study has found that the cytoprotective effect of C was stronger than that of R, and combining these two compounds with a higher proportion of C resulted in a synergistically enhanced cytoprotective effect and increased cell viability. The optimal composition of CR combinations with 8 : 2 indicated that C exhibited the dominant effect in this synergy of endothelial cryoprotection against oxidative stress, whereas R assisted to enhance the effect of C. Such mode of synergistic action may be attributed to the multitargets of C and R intracellularly.

Then, we investigated if these two compounds have a crosstalk in intracellular signaling pathways that contribute to the overall enhanced protective effect. A common molecular target that contributes to the endothelial protection of C and R is the activation of antioxidant Nrf2 [31, 36, 37]. The effect of R on Nrf2 and downstream HO-1 has been linked to its protective effect in pathological conditions such as acute ischemic stroke [36], heat stress, [38] acute liver injury [39] and ethanol-induced liver oxidative damage [40]. R was also found to attenuate cerebral ischemic injury in rats and alleviate myocardial toxicity *via* the induction of Nrf2 [41–43]. Herein, we have quantified the Nrf2 protein expression inducted by CR 8 : 2 in comparison to C or R and found that CR combination dramatically boosted the cellular production of Nrf2 protein which was significantly higher than C or R. This finding suggested that the interaction of these two compounds occurred in the Nrf2-related pathway and led to the enhanced Nrf2 production and contributed to the enhanced antioxidant defence as evidenced by the enhanced SOD and NAD activities shown in our study. Furthermore, our results showed that the CR 8 : 2 induced the translocation of Nrf2, further confirming the involvement of Nrf2 in the observed synergy. Previous studies suggested that C and R can activate Nrf2 through their actions on several upstream targets. The induction of Nrf2 by C has been linked to the activation of phosphorated extracellular-signal-regulated kinase 1/2 in the upstream, which then effectively reduced the ROS level and prevented cell damage [44]. R was found to activate Nrf2 through the dissociation of Nrf2-Keap1 binding which increased Nrf2 translocation *via* the stimulation of p38 mitogen-activated protein kinase and sirtuin 1/forkhead box protein O1 signaling pathways [45]. Thus, it is possible that different actions of C and R on their upstream targets contribute to the greater effect of CR combination in inducing Nrf2 translocation and overall Nrf2 expression. Additionally, our optimal combination of CR in inducing higher Nrf2 translocation and expression was at 8 : 2 ratio, whereas CR with the ratio of 1 : 1 showed a lower effect (data not shown). Thus, it is likely that C plays a dominant role in the observed synergy of CR combination with the activation of Nrf2. This mechanism may be implicated in other synergies of combinations/compounds or analogues that involve Nrf2 pathway. However, further study is needed to confirm this assumption.

Interestingly, the induction of Nrf2 by C was more significant than that of R, which has led to slightly higher HO-1 production. This is in line with their effects on ROS scavenging and cytoprotective effects as mentioned above. Neither C or R showed significant improvement of SOD or NAD production, although CR combination significantly increased SOD activity and NAD production. This increase may be related to the Nrf2-HO-1 mechanism. Thus, the induction of Nrf2 and the generation of antioxidant elements are likely to play a major role in the synergistic endothelial protection activity of CR, as the induction of Nrf2 has been demonstrated to launch the expression of enzymes that directly detoxify ROS including SOD and NAD+ [46–48]. However, multiple regulators are also involved in the production of SOD, mitochondria, and NADPH oxidase [47]. Further studies may explore the role of other regulators to explain the observed synergy such as nuclear factor*-κ*B and activator protein 1 which will help to elucidate the relevant mechanisms.

In addition, we have explored the combined activity of C and R in NAD coenzyme production in EAhy926 cells for the first time. In response to excessive oxidative stress, the poly adenosine diphosphate-ribose polymerase-1-dependent signaling in apoptosis is activated, which leads to NAD + depletion and then the release of apoptotic inducing factor and caspase-3-mediated cell death [49]. As a substrate, the restoration of NAD + levels is necessary for NADPH formation and sirtuin activity to induce cellular repair and stress resistance [50]. The publication by Howitz et al. reported that R activated sirtuin 1, the key regulator of NAD+, which is a mammalian NAD+-dependent protein deacetylase that promotes cell survival [51]. However, the action of resveratrol on NAD production in EAhy926 cells was not prominent in our study. Nevertheless, the NAD production was markedly increased in the CR 8 : 2. Such action may be related to the upregulated SOD enzyme, which affects sirtuin 1 and 3 and thus enhanced the activity of NAD+ [52].

## 5. Conclusions

The findings presented in this study suggested that C and R interacted synergistically in restoring cell viability in H_2_O_2_ impaired EAhy926 cells. The observed synergy of the optimal combination of CR 8 : 2 was likely to be attributed to the strengthened activity to reduce ROS and activate Nrf2, HO-1, SOD, and NAD regulators. Based on these results, we conclude that C and R combined in a certain composition can act as a more potent agent than the individual components in protecting vascular endothelium against oxidative stress, and the mechanism of this synergy is mediated at least partly by the activation of Nrf2-HO-1 pathway. Our findings advance the knowledge in developing new therapies for treating endothelial dysfunction against oxidative impairment.

## Data Availability

The analysed data used to support the findings of this study are included within the article, and the raw data used to support the findings of this study are available from the corresponding author upon request.

## Supplementary Materials

Supplementary Materials Supplementary Material 1: CI values of CRs at different Fa levels as assessed by CompuSyn based on the CI model, which reflected the synergistic interaction of C and R in restoring cell viability of EAhy926 cells against H_2_O_2_-induced cytotoxicity.
