# Supplementary material for: Synergistic Protective Effect of Curcumin and Resveratrol against Oxidative Stress in Endothelial EAhy926 Cells
Source: Evid Based Complement Alternat Med. 2021 Sep 3;2021:2661025. doi: 10.1155/2021/2661025 (PMC8434903; doi:10.1155/2021/2661025)
Supplement: Supplementary Materials — Supplementary Material 1: CI values of CRs at different Fa levels as assessed by CompuSyn based on the CI model, which reflected the synergistic interaction of C and R in restoring cell viability of EAhy926 cells against H2O2-induced cytotoxicity. [file 2661025.f1.docx]

## Supplementary Materials

Supplementary material 1. CI values of CRs at different Fa levels as assessed by compusyn based on CI model, which reflected the synergistic interaction of C and R in restoring cell viability of EAhy926 cells against H_2_O_2_ induced cytotoxicity.

| CRs | CI values at various Fa levels on Alamar blue assay | | | CI values at various Fa levels on MTT assay | | |
| --- | --- | --- | --- | --- | --- | --- |
|  | Fa_25_ | Fa_50_ | Fa_75_ | Fa_25_ | Fa_50_ | Fa_75_ |
| 1:9 | 0.42 | 0.27 | 0.23 | 30.09 | 1785.55 | 5.122E9 |
| 2:8 | 0.21 | 2.22 | 18.60 | 4.64E13 | 8.4E-10 | 3.3E-28 |
| 3:7 | 3118.85 | 1.71E18 | 1.12E33 | 3.09E10 | 1.59E-5 | 1.0E-16 |
| 4:6 | 0.08 | 0.03 | 0.01 | 4484.13 | 0.39 | 0.27 |
| 5:5 | 0.07 | 0.01 | 0.00 | 6107.54 | 0.49 | 0.21 |
| 6:4 | 0.02 | 0.00 | 3.21E-4 | 1554.77 | 0.24 | 0.14 |
| 7:3 | 0.03 | 0.01 | 0.00 | 2670.08 | 0.30 | 0.08 |
| 8:2 | 0.10 | 0.01 | 0.00 | 1915.61 | 0.46 | 0.15 |
| 9:1 | 0.10 | 0.07 | 0.03 | 15.71 | 0.98 | 36.93 |
